# Non-Epithelial Stromal Cells in Thymus Development and Function

**DOI:** 10.3389/fimmu.2021.634367

**Published:** 2021-02-25

**Authors:** Kieran D. James, William E. Jenkinson, Graham Anderson

**Affiliations:** Institute of Immunology and Immunotherapy, University of Birmingham, Birmingham, United Kingdom

**Keywords:** thymus, thymocyte development, mesenchyme cells, endothelial cell, lymphoid tissue development

## Abstract

The thymus supports T-cell development *via* specialized microenvironments that ensure a diverse, functional and self-tolerant T-cell population. These microenvironments are classically defined as distinct cortex and medulla regions that each contain specialized subsets of stromal cells. Extensive research on thymic epithelial cells (TEC) within the cortex and medulla has defined their essential roles during T-cell development. Significantly, there are additional non-epithelial stromal cells (NES) that exist alongside TEC within thymic microenvironments, including multiple subsets of mesenchymal and endothelial cells. In contrast to our current understanding of TEC biology, the developmental origins, lineage relationships, and functional properties, of NES remain poorly understood. However, experimental evidence suggests these cells are important for thymus function by either directly influencing T-cell development, or by indirectly regulating TEC development and/or function. Here, we focus attention on the contribution of NES to thymic microenvironments, including their phenotypic identification and functional classification, and explore their impact on thymus function.

## Introduction

The production of a diverse αβ-T-cell pool is vital to establishing and maintaining a functional adaptive immune system. While adult lymphoid progenitor cells are produced by the bone marrow, their lineage commitment and development into mature T-cells is dependent on their migration into the thymus, where essential interactions with heterogeneous thymic stromal cells take place (1). Arguably, the most recognized and well-studied stromal populations in thymus biology are thymic epithelial cells (TEC) within the cortical and medullary areas, which are defined by their anatomical separation and function. Cortical epithelial cells (cTEC) support the earliest thymocyte progenitor populations, guiding them through the cortex, directing them to a T-cell lineage fate and ensuring their functional qualities as self-MHC restricted cells through the process of positive selection (2). Medullary epithelial cells (mTEC), in conjunction with dendritic cells, then take over control of developing thymocytes. Through the process of producing and presenting a diverse array of self-antigens, mTEC drive single positive thymocytes either down conventional or Foxp3^+^ regulatory thymocyte lineages (3). In addition, mTEC screen thymocytes for their expression of high affinity αβ-TCRs, with negative selection limiting the release of these reactive T-cells into the peripheral pool (4). In addition to intrathymic selection, and during this medullary residency period, CD4^+^ and CD8^+^ single positive (CD4SP, CD8SP) thymocytes acquire the ability to proliferate in response to TCR stimulation, and undergo licensing for cytokine production, prior to exiting the thymus to join the peripheral T-cell pool as Recent Thymus Emigrants (RTE) (5, 6). While many studies have demonstrated the importance of cTEC and mTEC during T-cell development, thymic stromal microenvironments also contain heterogenous non-epithelial stromal (NES) populations in a similar manner to that seen in peripheral lymphoid tissues. Broadly separated into mesenchymal and endothelial cells, NES have been implicated in thymus organogenesis, thymocyte development, tolerance induction and development/maintenance of epithelial stroma. As with TEC, our understanding of NES has been improved through the phenotypic identification of new subpopulations, such studies on thymic mesenchyme and endothelium have provided new and important insight into their complexity and functional importance. In this review, we cover how the non-epithelial compartment of thymic stroma represent essential cell populations in regulating thymus function.

## Thymic Mesenchyme

### Mesenchyme in Early Thymus Development and Function

#### Organogenesis and Origin

In early experiments, physical separation of mesenchymal and epithelial stroma from embryonic murine thymus resulted in defective thymus development when epithelium was cultured *in vitro* in the absence of mesenchyme (7). These findings were confirmed in later studies in which removal of mesenchyme from embryonic day 12 murine thymic lobes impaired thymus growth *in vitro* (8). Furthermore, *in ovo* surgical ablation of cephalic neural-crest mesenchyme resulted in significantly reduced thymic size in fertile Arbor Acre chick embryos (9). Collectively, these studies provided clear evidence that the presence of mesenchymal stroma is key during the earliest stages of thymus development, when the endodermal-derived TEC rudiment is enveloped within and colonized by peri-thymic neural crest (NC)-derived mesenchyme. Moreover, studies by Jiang et al. (10) utilized Wnt1^Cre^ mediated fate mapping models to directly demonstrate the association of NC-derived mesenchymal cells with the embryonic murine thymus. Interestingly, although this study, and another by Yamazaki et al. (11) using a myelin protein zero fate mapping model, suggested that NC-derived mesenchymal cells could be scarcely detected in the postnatal thymus, subsequent studies using Wnt1^Cre^ based models provided evidence that NC-derived mesenchyme persists in adult thymic microenvironments (12, 13). Collectively, these findings in mammalian systems are consistent with the studies by Bockman and Kirby (9) using avian models that specifically pointed towards the importance of cells of neural crest origin in early thymus development.

With regard to the mechanisms of thymic mesenchyme function, their production of a number of growth factors has been shown to influence TEC populations. For example, Revest et al. discovered that fibroblast growth factor 7 (FGF7) and FGF10 produced by thymic mesenchyme and their receptor FGF-R2IIIb expressed by TEC were also essential for normal TEC development (14). In the absence of FGF-R2IIIb, thymus development appeared arrested around embryonic day 12.5, and the absence of either FGF-R2IIIb or FGF10 resulted in hypoplastic thymi and reduced TEC proliferation (14). In later studies, enzymatic removal of thymic mesenchyme was shown to reduce TEC proliferation (15), which could be restored by the addition of exogenous FGF7 and/or FGF10 *in vitro*. Interestingly, this study also found that while mesenchyme and FGF7/10 are essential for TEC proliferation, they are disposable for TEC differentiation, with embryonic TEC acquiring cTEC and mTEC phenotypes in the absence of mesenchyme and importantly these epithelial cells are functionally mature (15). Indeed, transplantation of embryonic day 12 thymus lobes stripped of thymus mesenchyme remained small, but retained the ability to support a complete program of T-cell development (16). Such findings suggest that an important property of thymic mesenchyme is to induce the proliferation of developing TEC in order to provide increasing numbers of intrathymic niches to support efficient T-cell development. In the absence of such mesenchyme-derived signals, niche availability remains limited and results in diminished T-cell production. In addition, epidermal growth factor (EGF) has been suggested to be a product potentially produced by thymic mesenchyme to support TEC proliferation and thymus lobule formation, as EGF can replace thymic mesenchyme in supporting TEC in cultures (17). Similarly, insulin-like growth factor (IGF) has been shown to be a mesenchyme product and IGF has been shown to regulate TEC development, but it has not been directly shown that mesenchymal-specific loss of either IGF or EGF leads to TEC defects (16–18). Thus, thymic mesenchymal cells play an important role in quantitatively regulating thymus function. While several mesenchymal growth factors have been implicated in this process (e.g., FGF7/10), further studies are required to examine the distinct functional properties, and cellular origins, of these molecules. Relevant to this, expression of the transcription factor MafB by thymic mesenchyme was shown to regulate production of Wnt3, Wnt11, and BMP4 which regulate TEC function, including the production of chemokines required for normal progenitor homing to the thymus (19).

Finally, as well as influencing the functional properties of TEC, mesenchyme has also been shown to influence the anatomic positioning of the thymus during its development. Studies of *Pax3^Sp/Sp^* mice, which carry a point mutation in Pax3 eliminating protein function, revealed that separation of the thymus from the pharynx, migration of the thymus to its position at the mediastinum and distribution of the thymus:parathyroid domains are dependent on the presence of thymic mesenchyme (17, 20). The thymus of these mice was significantly increased in size whereas the parathyroid was reduced and there was a delay in the separation of the two as a result of dysfunctional boundary formation between the thymus and parathyroid domains (20). Interestingly, evidence also suggests that a bi-directional relationship may exist between neural-crest derived mesenchyme and endoderm, where epithelial-derived FGF8 may regulate mesenchymal populations, where FGF8 hypomorphs demonstrate ectopic thymus positioning and thymic hypoplasia possibly as a result of impaired mesenchymal expression of FGF10 (21).

#### Mesenchyme and T-Cell Development

Using the reaggregate thymic organ culture (RTOC) technique, where defined thymocyte and stromal subsets can be reassembled into 3-dimensional structures, studies provided evidence that specific stages of thymocyte development are also dependent on the presence of thymic mesenchyme (22, 23). For example, culture of CD4^−^CD8^−^ (DN) T-cell precursors in the presence of TEC alone resulted in an absence of further T-cell development. In contrast, the addition of mesenchymal cells resulted in development to the CD4^+^CD8^+^ double positive (DP) and CD4SP and CD8SP stages (23). Importantly, TEC alone were able to support the development of CD4^+^CD8^+^ thymocytes to the CD4SP and CD8SP stages, indicating that a stage-specific requirement for mesenchyme operates at the DN-DP but not DP-SP transition. Later, further analysis revealed that the importance of thymic mesenchyme was specific to supporting the development of CD25^+^CD44^+^ DN2 thymocytes, with independence from mesenchyme occurring from the CD25^+^CD44^−^ DN3 stage (22). How thymic mesenchyme influences specific stages of T-cell precursor development remain unclear. However, it is interesting to note that this requirement can be met by both mesenchyme cells of non-thymic origin (eg embryonic lung) or fibroblast cell lines (eg NIH-3T3) (23). Moreover, and as demonstrated for bone marrow stroma (24), mesenchymal cells that regulate lymphoid progenitor development are effective presenters of the key cytokine IL7 *via* their production of extracellular matrix components (22), suggesting a possible mechanism for their involvement in early T-cell development. Importantly however, it is important to note that studies on the regulation of T-cell development by thymic mesenchyme are based largely on the requirements for fetal lymphoid progenitors, and often involve analysis of T-cell development using *in vitro* culture. As such, further studies are required to assess whether similar requirements operate in both the fetal thymus *in vivo*, and whether DN T-cell precursors in the adult thymus are similarly controlled by mesenchyme.

### Mesenchyme in the Adult Thymus

#### Characterization

While initial studies suggested that NC-derived thymic mesenchyme does not persist beyond the embryonic thymus (10, 11), further studies utilizing NC specific Wnt1^Cre^ and Sox1^Cre^ mouse models (crossed to Rosa26 reporter mice) enabled identification of NC-derived mesenchymal cells within adult thymus (12, 13). Here, thymic mesenchyme is located in multiple places. It helps contribute to the structure of the thymus lobe making up the capsule around the outside of the thymus (25, 26) and thymic mesenchyme has been reported in the cortex (13), medulla (26) and as well as around blood vessels both dispersed throughout the thymus, and at the corticomedullary junction (CMJ) (27–29). In addition, Komada et al. demonstrated that while NC-derived mesenchyme contributes to pervivascular cells, mesoderm-derived cells contribute to endothelial compartments (30). Thus, mesenchymal cells are present in the adult thymus at multiple sites where distinct stages of T-cell development take place. In addition to anatomic location, various phenotypic markers have been used to identify thymic mesenchymal cells, and different studies have used various marker combinations to define thymic mesenchymal stroma and subsequent subpopulations. Currently, a clear consensus on the panel of mesenchymal markers to be used is lacking, and for markers that are used, there is often overlap in cell populations that are studied. For example, commonly used markers of thymic mesenchyme include PDGFRα, PDGFRβ, Ly51, Podoplanin (Pdpn, gp38) MTS-15, ERTR7, and FSP1 (13, 16, 25, 27, 31–33). Pdpn is a notable marker, as it is frequently used to identify thymic mesenchyme and often stained alongside CD31 to distinguish between endothelium and mesenchymal cells, an approach also used in peripheral lymph nodes to identify the fibroblastic reticular, lymphatic endothelial and blood endothelial cell subsets (29, 34). Interestingly, in addition to being utilized as a phenotypic marker of mesenchymal populations, Pdpn may potentially play functional roles in the thymus. For example, Pdpn has been proposed to contribute to CCL21 localization in thymic microenvironments and contribute to the development of regulatory T cells in young mice (35). It is important to note that while Pdpn and CD31 expression can highlight some similarities in the populations of non-epithelial stroma, unlike peripheral secondary lymphoid tissues where a clear lymphatic stromal population is present, the presence/absence of lymphatics within the thymus has been difficult to resolve. Previous confocal analysis of the thymus to detect expression of VEGFR3 and LYVE-1 suggested the presence of lymphatic vessels (25). Using Lyve-1^Cre^–mediated fate mapping, or dual analysis of CD31/Pdpn expression by flow cytometry, lymphatics were extremely rare and difficult to observe (29). Use of Lyve1^Cre^ to target any potential lymphatic population within the thymus is complicated by the observation that this approach labels a significant proportion of thymocytes when crossed to a reporter model (36).

Recent phenotypic analysis of thymic mesenchyme from Sitnik et al. revealed Pdpn^+^ and Pdpn^−^ populations within PDGFRβ^+^ thymic mesenchyme in the adult thymus (27). The inclusion of the additional markers CD34 and Itgα7 was further used to identify these two subsets as Pdpn^+^CD34^+^ adventitial mesenchyme cells and Pdpn^−^Itgα7^+^ thymic pericytes, where adventitial mesenchyme surrounds pericytes which in turn surround blood endothelium of vessels (27, 28). A recent advancement has shown that the marker Dipeptidyl-peptidase 4 (DPP4) can be used to distinguish between capsule fibroblasts (capFb, which are DPP4^+^) and medullary fibroblasts (mFb, which are DPP4^−^), further characterizing the different mesenchymal populations present in the adult thymus (26). How these DPP4^+^ mesenchymal subsets relate to CD34^+^ adventitial and Itgα7^+^ pericytes described previously is not clear. Thus, follow up studies are important to examine how various definitions of mesenchyme populations correlate with each other, in order to provide a clearer stratification of subset heterogeneity that would allow effective analysis of thymic mesenchyme function in the adult thymus.

### Functional Roles of Adult Thymic Mesenchyme

#### Regulation of Thymic Epithelial Cells

While mesenchymal cells regulate the proliferation of embryonic TEC (14, 16), studies demonstrate that thymic mesenchyme can also be a negative regulator of TEC expansion during embryogenesis and that this property is maintained in the adult thymus (32). Thus, thymic mesenchyme was identified as a major intrathymic source of retinoic acid (RA) which was found to restrict TEC proliferation, in particular within the cTEC compartment (32). Interestingly, these same cells that produce RA may also have the ability to produce growth factors such as FGF7/10 that have a positive impact on TEC proliferation during thymus organogenesis and perhaps have a continued role in the adult thymus (14–16). Thus, thymic mesenchyme has the ability to both positively and negatively regulate the TEC compartment in the developing and adult steady-state thymus. Interestingly, thymic mesenchymal cells from adult thymus have also been shown to be capable of sphere-formation, a property initially thought to define TEC stem cells. Thus, Sheridan et al. (37) showed that thymosphere-forming cell (TSFC) represent long-lived cells of mesenchymal origin which also possess progenitor-like capacities, and are able to give rise to adipocytes (37). While, the functional properties of sphere-forming mesenchymal cells require further examination, evidence of a role for thymic mesenchyme in regulating TEC subsets was reported in studies where mesenchyme-specific deletion of LTβR was achieved using Twist-2^Cre^ mice (26). Here, deletion of LTβR on thymic mesenchyme resulted in a number of intrathymic changes, including reduced mTEC numbers in the adult thymus (26). Whether this is indicative of mechanism where thymic mesenchyme regulates TEC in the steady state adult thymus, or rather relates to the role of mesenchyme regulating TEC during thymus organogenesis requires further examination. Similarly, thymic fibroblasts defined by Fibroblast specific protein 1 (FSP1) expression appear to be an important regulator of TEC populations (33). Indeed, FSP1^+^ mesenchyme produce IL-6 and FGF7, and the absence of FSP1^+^ mesenchyme results in a significantly smaller thymus size and reduced TEC numbers (33). Interestingly, as well as being a useful phenotypic marker, FSP1 itself may be an important molecular mediators of adult thymus mesenchyme function, as addition of FSP1 to TEC in culture significantly increases their proliferation and expression of CD80 and AIRE (33).

Collectively, the studies above highlight how thymic mesenchymal stroma supports the TEC compartment in the adult thymus at steady state. Additionally, some findings also implicate thymic mesenchyme in supporting TEC and thymus recovery following damage. For example, Sun et al. found that deleting FSP1^+^ thymic mesenchyme resulted in significantly delayed regeneration in a cyclophosphamide-induced thymic involution model (33). Gray et al. also found that upon cyclophosphamide-induced thymic involution, MTS-15^+^ thymic fibroblasts expanded during regeneration and produced FGF7, FGF10 and IL-6 to promote TEC proliferation and increase T-cell production (31). Interestingly Sun et al. found that FSP1^+^ mesenchyme was distinct from MTS-15^+^ fibroblasts thus demonstrating some conservation of function between the two mesenchymal populations (31, 33). These are distinct from MTS-15 expressing thymic mesenchyme as shown by FACs but also by confocal showing these cells were localized in different areas of the thymus (33). Finally, expression of CD248 (endosialin), a protein linked to remodeling of tissues, is induced within PDGFRα^+^ thymic mesenchyme during infection (38). In a Salmonella infection model, CD248-deficient mice exhibited reduced thymus size and poor thymus regeneration, indicating a specific but poorly understood role for CD248 expression by mesenchyme in controlling the recovery of thymus function following acute thymus atrophy (38).

#### Thymus Emigration

The microanatomical positioning of adventitial cells and pericytes around blood vessels in adult thymus, suggest a possible role in regulating the egress of mature thymocytes from the thymus following their intrathymic development. The sphingosine-1-phosphate (S1P) pathway has been shown to be a key axis for the egress of mature thymocytes (29, 39, 40), with a complex interplay of multiple cellular compartments, including thymic mesenchymal cells being involved in this pathway. Regulation of the S1P pathway centers on maintaining a low gradient of S1P within the thymic parenchyma and restricting availability to points of exit, *via* the production of S1P *via* kinases and transporters, or the degradation of S1P (which can be by reversible and non-reversible means) (41). The sphingosine kinases SPHK1 and SPHK2 are essential regulators of S1P production, where deletion of *Sphk1* in NC-derived thymic mesenchyme, using conditional Wnt1^Cre^ mediated deletion, disrupts thymocyte egress (29). Mature SP in the thymus of these mice had elevated levels of S1PR1 expression that is consistent with reduced exposure to available S1P. These findings were accompanied by an intrathymic accumulation of mature SP thymocytes, a phenotype highly indicative of an egress defect (29). Mesenchyme can also influence the S1P gradient through S1P lyase, an enzyme which irreversible cleaves S1P and thus in the context of egress acts to maintain low intrathymic S1P levels and ensure migration of mature thymocytes towards high concentrations of S1P present in blood (39). Consistent with this role, it has been shown that S1P lyase expression is focused around blood vessels where it is expressed by thymic endothelial cells as well as thymic mesenchyme (39). The importance of S1P lyase in regulating egress was highlighted by the use of the S1P lyase inhibitor ﻿2-Acetyl-4-tetrahydroxybutylimidazole (THI), which causes an intrathymic accumulation of mature SP thymocytes and reduced T-cell output (39, 42). Based on the location of its expression and its mode of action the authors suggest that S1P lyase degrades S1P in the PVS irreversibly to create a low S1P environment which again acts to promote the egress of mature thymocytes (39).

In addition to the S1P pathway, mesenchyme regulates thymic egress through an additional pathway that includes the chemokine receptor CCR7, a pathway previously shown to play an important role in neonatal thymus egress (43, 44). We showed that in the absence of both CCL21 and CCL19, thymic egress of conventional SP thymocytes is impeded, with this requirement specifically mapping to a non-redundant role of CCL21 but not CCL19 (28). In the adult thymus it is known that CCL21 is produced by an mTEC subset, as most recently shown through the use of CCL21^TdTom^ reporter mice (45). Interestingly, analysis of CCL21 protein distribution in the neonatal thymus revealed this was concentrated around blood vessels at the corticomedullary junction (28). Further analysis revealed that expression of heparin sulfate by CD34^+^ adventitial cells, and to a slightly lesser extent expression by Itgα7^+^ pericytes, regulated CCL21 presentation by these mesenchymal populations found at this site (28). Thus, while CCL21 is a product of mTEC, its presentation is dependent on thymic mesenchymal populations for neonatal thymic egress, highlighting the combined action of epithelium and mesenchyme for regulation of a key process in thymocyte development. It is important to note that the role of CCL21 in egress is limited to the neonatal window, as adult CCR7-deficient mice show no thymic egress defect (44, 46). Thus, thymic mesenchymal regulation of thymocyte egress is temporal and limited to the neonatal window and thus the earliest waves of αβ thymocyte egress (28). The precise differential impact of CCL21 on thymocyte emigration at neonatal and adult stages remains unclear. Interestingly however, previous studies have suggested potential differences in the expression of the S1P receptor S1P1 in neonatal versus adult SP thymocytes (47). Evidence from this study demonstrated that neonatal thymocyte egress can be blocked using FTY720 treatment, indicating that the S1P-axis is functional in the window when S1P1 expression is low and that CCL21 also regulates thymus egress, raising the interesting notion that CCL21 may act to compensate for or facilitate reduced S1P- axis activity in the perinatal period, with this requirement being superseded by increased S1P1 expression at adult stages.

Additional studies have further proposed that expression of the lymphotoxin β receptor (LTβR) by specific thymic stromal compartments, including mesenchyme, may regulate thymocyte egress in the adult thymus. Germline LTβR deletion leads to an intrathymic accumulation of mature thymocytes and reduced recent thymic emigrants (RTE) being indicative of a thymic egress defect (48, 49). In a Twist-2^Cre^ x *Ltbr* ^Flox^ mouse model, where LTβR deletion is restricted to thymic mesenchyme, Nitta et al. additionally noted an intrathymic accumulation of mature SP thymocytes suggesting that the egress defect seen in germline LTβR-deficient mice could potentially include a requirement for LTβR on thymic mesenchyme, however increased intrathymic numbers of SP thymocytes could be a consequence of reduced negative-selection observed in these mice (26). In other models aimed at deletion of LTβR from mesenchyme (Wnt1^Cre2^xLTBR^Flox^), this SP thymocyte accumulation and emigration defect was not observed. While the reasons for this difference are not clear, it may be that while both Wnt1^Cre2^ and Twist2^Cre^ are active in thymic mesenchyme, the exact subsets that are targeted differ. It will be important to formally define the types of thymic mesenchyme that are targeted in specific Cre expressing mouse strains in order to gain a clearer picture of the role of individual mesenchyme subsets controlling T-cell development.

#### Thymic Tolerance

In line with the abundance of mesenchymal stroma within medullary areas, these cells have also been implicated in the regulation of tolerance induction of developing thymocytes. Interestingly this finding was perhaps born from attempts to understand how signaling *via* LTβR within TEC microenvironments influenced thymocyte development. First described by Boehm et al., 2003, mice deficient for LTβR exhibited a clear reduction in mTEC numbers, and severely disrupted medullary organization (48). Instead of characteristic large medullary areas surrounded by cortex, thymus from LTBR-KO mice had an increased frequency of smaller medullas dispersed throughout the organ (48, 50). These mice also displayed intrathymic accumulations of mature SP thymocytes indicating disrupted thymocyte egress, as well as autoimmune symptoms including autoantibodies and lymphocytic infiltrates into tissues (48, 50, 51).

Through the use of a panel of stromal-cell specific Cre mice crossed to LTβR^Flox^ mice, we examined the contribution of epithelium, mesenchyme and endothelium to regulation of LTβR-dependent tolerance induction in the thymus (50). Deletion of *Ltbr* on TEC recapitulated the mTEC phenotype of *Ltbr* ^−/−^ mice, but despite this, there was no autoimmune or T-cell egress defect in these mice (50, 51). *De novo* Treg generation was found to be independent of LTβR signaling and instead it appeared that dendritic cells were also disrupted in the LTβR-germline KO thymus (50). Screening of different stromal cell specific *Ltbr* deletion models revealed that only when *Ltbr* is deleted on Wnt1^Cre2^-expressing thymic mesenchyme (LTβR^Mes^), identified as CD45^−^EpCAM-1^−^CD31^−^Pdpn^+^ by flow cytometry, was the DC-defect of *Ltbr* ^−^ *^/^* ^−^ mice replicated and this pathway was proposed as the mechanism by which LTβR signaling regulates thymic tolerance (50). Further analysis of DC kinetics revealed an essential role for CCR7 expression by DC and CCL21 production by thymus for development of Sirpα^−^ conventional DC type 1 (cDC1), a population significantly reduced in *Ltbr* ^−^ *^/^* ^−^ and LTβR^Mes^ mice (52). Thus, it is interesting to speculate whether the role of CCL21 presentation by thymic mesenchyme is LTβR-dependent and the possible mechanism that causes the tolerance breakdown in *Ltbr* ^−^ *^/^* ^−^ thymus. Interestingly it has been shown that thymic DC can regulate thymocyte egress *via* S1Plyase (53). However, LTβR^Mes^ mice, utilizing the Wnt1^Cre2^ promotor, do not exhibit the egress defect seen in *Ltbr* ^−^ *^/^* ^−^ mice, suggesting that the LTβR-dependent regulation of thymic DC is not essential for thymocyte egress (50, 51).

Using Twist2^Cre^LTβR^Flox^ mice Nitta et al. showed that despite no observed DC defect, there were increased auto-antibodies and cellular infiltrates and this correlated with reduced numbers of Pdpn^+^ thymic mesenchyme which lacked mFb-specific genes including specific TRAs (26). They suggest that expression of MHC Class I, in conjunction with evidence of protein handover/pickup by thymic DC, work to support central tolerance induction during thymocyte development and thus thymic mesenchyme has a significantly more direct role in thymus tolerance *via* self-Ag production rather than regulating DCs (26). Interestingly, this pathway would appear to mirror that proposed for regulation of peripheral tolerance by non-epithelial fibroblastic reticular stromal cells in secondary lymphoid tissues (54). The striking difference here between the two mesenchyme-specific models for deleting *Ltbr* is that while there is a clear DC defect in Wnt1^Cre2^LTβR^Flox^ mice used in the Cosway et al., study there is no egress defect, whereas in the Twist2^Cre^LTβR^Flox^ of the Nitta et al., study the reverse is true (26, 50). As the Cre-lines used in each study appear faithful it remains difficult to fully elucidate the exact role that LTβR and thymic mesenchyme is playing in regulating thymic tolerance or thymic egress. A follow up of Nitta et al. study to understand how their data of an Ag presentation role of mFbs fits into the landscape of CD34^+^ adventitial and Itgα7^+^ mesenchyme, and what further gene expression differences exist within these mesenchyme populations would be very interesting and useful to further our understanding of the heterogenous populations and roles that thymic mesenchyme in the adult thymus carry out.

## Thymic Endothelium

### Regulation of Thymocyte Development

#### Thymus Colonization by Lymphoid Progenitors

**Figure 1:**
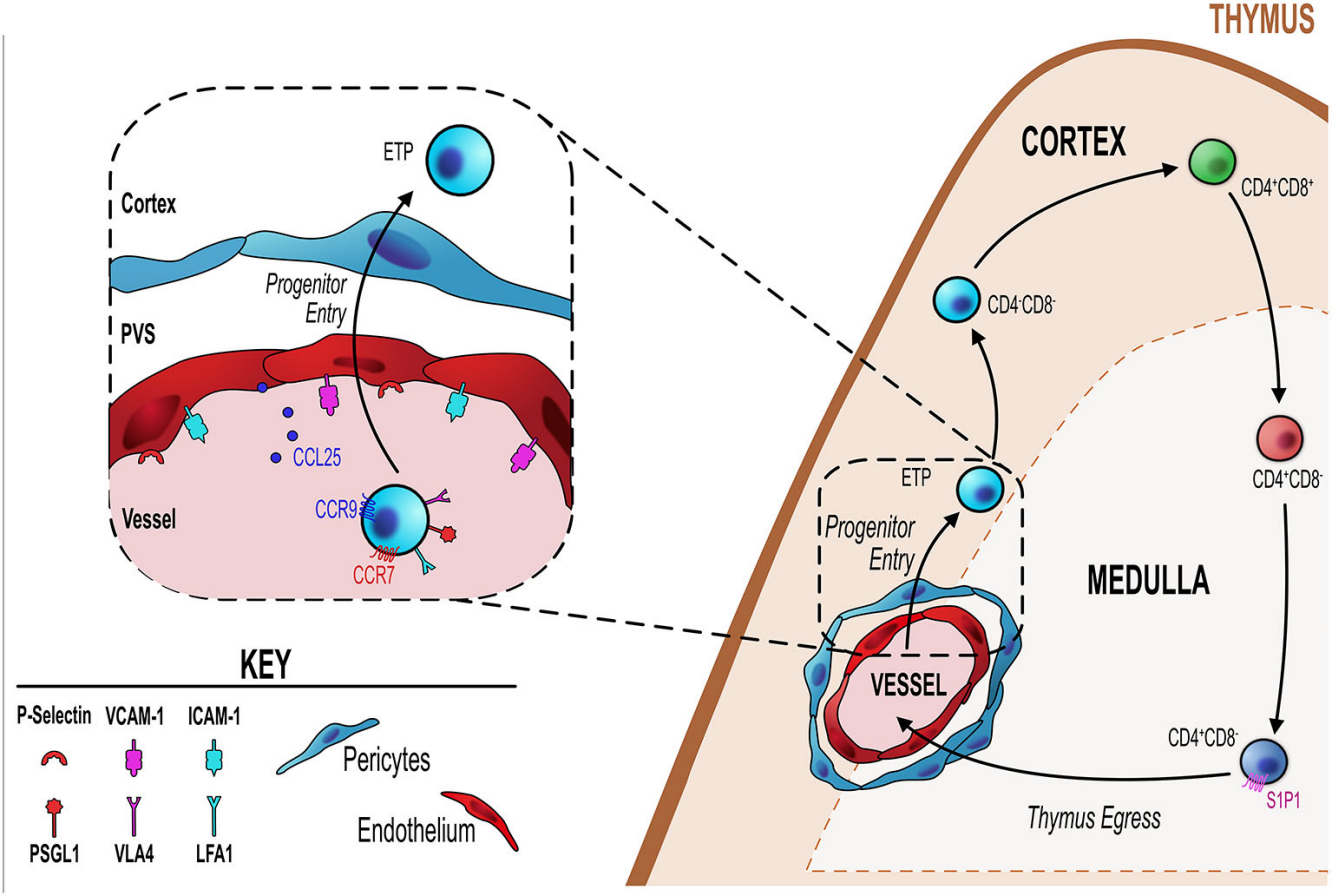
Endothelial cells regulate T-cell progenitor entry. Initial steps in thymocyte development involve homing to, and colonizing of, the thymus by bone marrow-derived blood-borne progenitors *via* blood vessels at the corticomedullary junction (orange dotted line). Entry of these progenitors is controlled by thymic endothelial cells (EC) which regulate this process through a number of different mechanisms detailed in the inset figure (black dotted line/box). ECs express P-selectin, the ligand for which (P-selectin glycoprotein ligand 1, PSGL-1) is expressed by lymphoid progenitor cells. P-selectin-PSGL1 interactions are essential in slowing down progenitor movement, allowing them to interact with ICAM-1 and VCAM-1 expressed by ECs. In adult thymus, CCL25 (ligand for CCR9) which is produced by both TEC and ECs has also been shown to be essential for progenitor homing to the thymus. Additionally, CCR7 is involved in thymus colonization, although its precise ligand requirements (CCL19 and/or CCL21) is not known.

The thymus does not contain a pool of self-renewing hematopoietic stem cells and so relies on remote colonization of bone-marrow derived progenitor cells from the blood into the thymus. Thus, thymic endothelial cells play an essential role in interacting with the T-cell progenitors in the blood to ensure site-specific extravasation of progenitor cells into the thymus for the ongoing production of T-cells (55) (**Figure 1**). During development, thymus vascularization is evident around embryonic day 15.5 (E15) (56), with CD31^+^ endothelial cells abundant in thymus sections at E15.5, but not at earlier time points (56–58). The dense 3-D vascular network present at this stage was shown using an elegant approach in which intravenous microinjections of resin into embryos was performed, with subsequent digestion of tissues to leave a resin cast which was then imaged using scanning electron microscopy (56). It is interesting to note that T-cell progenitors colonize the thymus at earlier time points prior to thymus vascularization. At earlier stages (<E15) T-cell progenitors have to migrate through the perithymic mesenchymal layer that invests the early epithelial rudiment at this early stage in thymus organogenesis to enter epithelial microenvironments (56, 59, 60). During this period the CCR9 ligand CCL25 produced by Foxn-1–dependent thymic primordium and the CCR7 ligand CCL21 produced by Gcm2-dependent parathyroid, are essential in progenitor colonization of the early thymus (56, 61, 62). CXCR4 also aids colonization of the embryonic thymus, but the precise intrathymic cellular source is not clear (63). Critically, the requirement for CCR7 and CCR9-dependent thymus colonization is maintained into adulthood, where CCR7/CCR9 double deficient mice (*Ccr7* ^−^ *^/^* ^−^ *Ccr9* ^−^ *^/^* ^−^) demonstrate reduced thymus colonizing cells, albeit such reductions are potentially made up for by subsequent compensatory expansion of thymocytes (64).

A key endothelial mechanism that regulates T-cell progenitor entry into the thymus is the interaction between endothelial expressed P-selectin and P-selectin glycoprotein ligand 1 (PSGL-1)–bearing progenitor (65–67). PSGL1-deficient thymi have reduced numbers of ETP due to the reduced ability of progenitor cells to enter the thymus (66). Parabiotic or adoptive transfer experiments revealed clear evidence for the essential role of P-selectin:PSGL1 interactions for progenitor homing to the thymus (66, 67). Consistent with this, treating cells with anti-P-selectin neutralizing antibody significantly impaired progenitor homing to the thymus (67). This P-selectin/PSGL-1 interaction slows down progenitors allowing them to interact with ICAM-1 and VCAM-1, whose expression by the endothelium is also key in the process of progenitor homing and entry into the thymus (67). In a related study, Gossens et al. found that in addition to P-selectin, endothelial production of CCL25 is also be a key regulator of T-cell progenitor homing to the adult thymus (65). They found that mouse models with T-cell progenitor intrinsic entry defects were significantly more receptive to the homing and entry of WT T-cells progenitors (65). This increased receptivity correlated with increased P-selectin and CCL25 expression by thymic endothelial cells, suggesting both are involved in T-cell progenitor homing and colonization (65). Interestingly this same study revealed that endothelial expression of P-selectin and CCL25 is the mechanism by which endothelial cells enforce the temporal, gated, nature of thymus colonization, wherein the thymus goes through periods of receptiveness and non-receptiveness to colonization by new T-cell progenitors (55, 65, 68). This phenomenon may be part of a process by which progenitor niche availability within the thymus directly feeds back and acts on the ability of progenitors to enter the thymus or not (65, 69). Gossens et al. showed that progenitors act on the endothelium to limit the expression/production of P-selectin and CCL25, when the intrathymic population of progenitors is reduced and thus niche availability is increased, endothelial cells are signaled to increased P-selectin and CCL25 (65). This then drives increased progenitor entry, which fills the intrathymic niches signaling endothelial cells to reduce their production of P-selectin/CCL25 and the cycle continues (65). In line with this, P-selectin and CCL25 expression levels correlated with phases of thymic T-cell progenitor receptivity and non-receptivity (65). Additionally, in mouse models which have a significant T-cell progenitor reduction in the thymus and subsequently have increased niche availability, these mice showed increased expression of P-selectin and CCL25 and also increased receptiveness to peripherally induced WT T-cell progenitors (65). Therefore, not only do thymic endothelial cells regulate the entry of progenitor cells by their expression of key molecules such ICAM-1, VCAM-1, P-selectin and CCL25, but they act as gatekeepers regulating the phases of entry and no-entry to new progenitor cells by adjusting the amount and periodicity of their expression of CCL25/P-selectin (65). The mechanism that regulates these changes in expression of key genes is not clear. However, an interesting study highlighted a key role for the transcriptional regulator early growth response gene 1 (Egr1) as part of a negative feedback loop in this system. Here, Schnell et al. showed that Egr1-deficient mice had increased thymocyte number as a direct result of increased T-cell progenitor homing and interestingly Egr1 was expressed by DN thymocytes (70). Correlating with increased progenitor homing, they found that endothelial cells of Egr1-deficient thymus had increased P-selectin expression, suggesting that Egr1 mediates a feedback mechanism whereby the number of DN thymocytes controls the entry of new T-cell progenitors by regulating P-selectin expression by the thymic endothelium (70).

When T-cell progenitors enter the thymus *via* blood vessels, they do so *via* large venules found at the corticomedullary junction (CMJ), after which they migrate through the cortex towards the subcapsular zone (SCZ) to begin the step-wise development program within the thymus (71, 72). It has been shown that a subset of endothelial cells appears enriched at the CMJ and such cells play key roles in regulating T-cell progenitor entry. Shi et al. describe 3 subpopulations of CD31^+^ thymic endothelial cells based on their expression of Ly6C and P-selectin (73). Thymic portal endothelial cells (TPEC) were identified as Ly6C^−^P-selectin^+^ and were found to be enriched in vessels at the CMJ, with ~60% of CD31^+^ vessels at the CMJ found to contain TPEC (73). Importantly, TPEC are selectively reduced in mice lacking LTβR either in all cells, or in models were LTβR is deleted specifically on endothelial cells using a endothelial-specific Cre mouse model (Tie2^Cre^) crossed with LTβR^Flox^ mice, to generate LTβR^Endo^ mice (49, 73). Loss of TPEC in these mice is accompanied with a significant reduction in the number of early T-cell progenitors (ETP) within the thymus, indicating a T-cell progenitor homing/colonization defect (73, 74). Despite reduced ETP in the thymus of LTβR-deficient mice, thymocyte development is rescued by compensatory expansion of DN3 thymocytes which may expand due to the increased niche availability (69, 74). Interestingly, levels of ICAM-1 and VCAM-1 were noticeably reduced in thymic endothelial cells from LTβR-deficient mice, a finding consistent with a previous study showing that inhibition of ICAM-1 or VCAM-1 reduces thymus homing of progenitor cells (67, 74). The localization of TPEC at the CMJ and reduced ETP strong supports the proposed role of these cells in regulating progenitor entry (73, 74). Interestingly, in addition to a requirement for LTβR in the development of TPEC, absence of αβ-T-cells (as seen in TCRα^−/−^ mice) also significantly reduced the TPEC population, suggesting that provision of LTβR ligands by SP thymocytes may act as part of a crosstalk mechanism between thymocytes and endothelial cells to regulate development of the latter (73).

#### Thymocyte Development

In addition to regulating the entry of lymphoid progenitor cells, thymic endothelial cells have also been shown to regulate the development of downstream thymocyte populations. For example, Kit ligand (KitL) is a key regulator of early thymocyte proliferation and differentiation (75), with membrane-bound KitL (mKitL) being expressed by both cTEC and vascular endothelial cells found within the cortex (76). Deletion of mKitL specifically on endothelial cells (using Tie2^Cre^PDGFRβ^Cre^ endothelial specific mice) resulted in a significant reduction in the frequencies of both ETP and DN1 thymocytes (76). In thymus sections, while DN1 thymocytes are in close proximity to mKitL producing endothelial cells (76), endothelial-derived mKitL was dispensable for T-cell development, suggesting the involvement of additional mKitL producing cells, such as cTEC, in control of ETP/DN1 progenitors (76). Interestingly it has been found that this mKitL-c-Kit interaction is bi-directional, with mKitL signaling leading to increased endothelial cell proliferation and thus endothelial mKitL is essential in both ETP and endothelial cell regulation (77).

**Figure 2:**
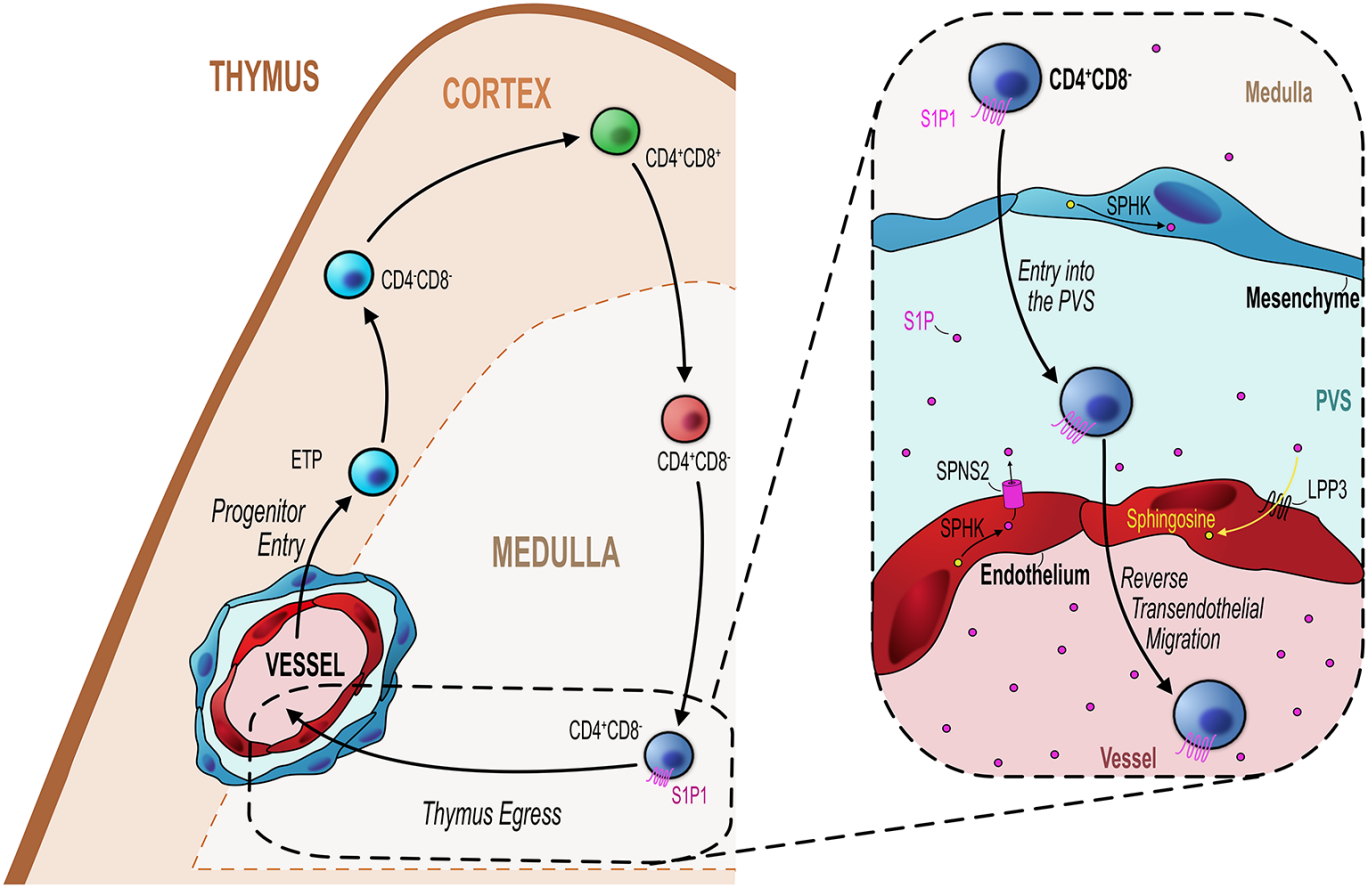
Endothelial cells regulate mature thymocyte egress. Similar to progenitor entry into the thymus, egress of mature thymocytes that have completed their intrathymic development occurs *via* blood vessels at the corticomedullary junction (orange hashed line). Mature CD4^+^CD8^−^ (SP4) or CD4^−^CD8^+^ (SP8) thymocytes which express the S1P receptor S1P1 migrate from the medulla to the CMJ to begin their exit from the thymus. As detailed in the inset figure (black dotted line/box), mature SP thymocytes first enter the perivascular space (PVS) and then cross the endothelium into the blood *via* reverse transendothelial migration, with both processes being S1P-dependent. Thymic ECs play an essential role in this process by maintaining the S1P gradient within the thymic parenchyma/PVS. ECs can increase S1P levels through their expression of the S1P transporter Spinster homolog 2 (SPNS2) which transports S1P synthesized through the phosphorylation of sphingosine by sphingosine kinase (SPHK). Conversely, ECs reduce S1P levels through expression of lipid phosphate phosphatase 3 (LPP3) which dephosphorylates S1P to sphingosine or by irreversibly degrading S1P *via* S1P lyase. These mechanisms maintain the export of mature thymocytes essential to contribute to the peripheral T-cell pool.

In addition to regulating the thymic entry, endothelial cells also play a key role in regulating the egress of mature thymocytes from the thymus into the blood (**Figure 2**). As discussed previously, the sphingosine-1-phosphate (S1P) pathway is an essential mechanism regulating egress of mature thymocytes from the adult thymus (78). Maintenance of localized intrathymic levels of S1P is important to ensure optimal egress and as such this is regulated by the balance of production or inhibition of S1P by particular cell types in the thymus. Endothelial cells have the capacity to achieve this by their expression of particular enzymes or transport molecules allowing them to influence the S1P gradient. For example, S1P lyase is one particular regulator of this process, and as well as being expressed by thymic mesenchymal cells, endothelial-specific expression of S1P lyase is also essential for egress (39, 42). Furthermore, endothelial cells express lipid phosphate phosphatase 3 (LPP3), a class of S1P-degrading enzyme which dephosphorylates S1P (79). Much like S1P lyase, its role in thymocyte egress is to help maintain low levels of S1P, but unlike S1P lyase this process is not irreversible, as LPP3 works to dephosphorylate S1P reverting it to sphingosine which is inactive to S1P1 (42, 79, 80). By deleting LPP3 in various cell types in the thymus, Breart et al. demonstrated the essential role of this enzyme in regulating thymocyte egress (79). Cell specific deletion of *Lpp3* through use of VE-Cadherin^Cre^ to target endothelial cells resulted in the intrathymic accumulation of mature SP thymocytes suggesting a block in thymocyte egress (79). Interestingly deletion of *Lpp3* in TEC using K14^Cre^ also caused an intrathymic accumulation, suggesting that both populations play non-redundant roles in regulating thymocyte egress *via* manipulating/maintaining the S1P gradient (79).

The expression of these two enzymes are examples of how endothelial cells can downmodulate levels of S1P. However, endothelial cells may also directly contribute to S1P availability by virtue of their expression of the S1P transporter spinster homolog 2 (*Spns2*) (81). Deletion of *Spsn2* in endothelial cells using a Tie2^Cre^ crossed to *Spsn2* ^Flox^ mouse model resulted in the intrathymic accumulation of SP thymocytes and reduced T-cell numbers in the periphery, indicating a severe T-cell egress defect (81). Follow up studies to understand these mechanisms in light of the more recent endothelial population definitions would significantly aid our understanding of how endothelial cells regulate egress *via* the S1P pathway. For instance, the localization of TPEC at the CMJ would make them an attractive candidate to regulate T-cell egress *via* these mechanisms. Perhaps in line with this, in the LTβR^Endo^ mouse model which have a T-cell progenitor homing defect, there is also clear indication that thymic egress is also LTβR-endothelial dependent (49). Thus, LTβR^Endo^ mice exhibit an intrathymic accumulation of mature thymocytes at a similar magnitude as seen in the germline LTβR-deficient mouse thymus (48, 51). As stated previously, these mice lack TPEC, suggesting these cells regulate both entry and exit of T-cell populations in an LTβR dependent manner (51, 73, 74). Interestingly, this endothelial-regulated stage of egress was restricted to the PVS-entry step, the penultimate step in the process of cells leaving the thymus (29, 39, 72, 82). Moreover, short-term FTY720 treatment prevents entry of mature T-cell in the PVS which may suggest that this process is S1P dependent; however; the exact mechanisms by which endothelial cells regulate thymus egress in an LTβR-dependent process remains unclear.

It has also been suggested that a thymus:blood barrier exists in the thymus (83). However, this idea is at odds with the fact that many of the vessels within the thymus function to allow the trafficking of progenitors and mature T-cells into and out of the thymus. Heterogeneity amongst thymic blood vessels indicates that different vessels have different permeabilities and provides evidence of how a thymus:blood barrier may be present while also serving as entry and exit points of the thymus. It has been shown that all cortical vessels express Cld5 but only half of the blood vessels in the medulla and CMJ express Cld5 (84). Thymus sections of mice injected intravenously with a biotin tracer revealed that the biotin tracer leaked into the medulla *via* Cld^−^ vessels whereas little penetration was found around Cld5^+^ vessels (84). The importance of Cld5 expression by cortical vasculature in maintaining their impermeable status was highlighted in experiments where Cld5 deletion led to cortical vessels becoming leaky (84). It is likely the leaky nature of medullary/CMJ vessels aids entry/exit of trafficking cells, consistent with these vessels being located at/near the CMJ. This is supported by findings of accumulations of mature thymocytes within the PVS of Cld5^−^ vessels and also by IV injection of tetramethylrhodamine-conjugated S1P which was found to leak into the medulla in thymus sections of injected mice likely contributing to the S1P-gradient required for thymus egress (84).

### TEC Regulation by Thymic Endothelium

Endothelial cells have also been reported to regulate TEC in the steady-state and during thymus regeneration. For example, Choi et al. showed that *via* Tie2^Cre^ mediated deletion of plexinD1 on endothelial cells, thymus lobes displayed reorganization of vasculature, with a significant increase of vessels in the subcapsular zone and a corresponding disruption of medulla localization, where medullary areas were found at the subcapsular zone and fused with the capsule (85). Thus, plxnd1 expression by thymic endothelial cells influences medullary formation within the thymus (85). Upon damage *via* total body sublethal irradiation, endothelial cells upregulate their production of bone morphogenetic protein 4 (BMP4) which in turn upregulates Foxn-1 expression in TEC, leading to increased expression of downstream Foxn-1 targets such as delta-like ligand 4 (DLL4) which are involved in TEC development, maintenance and regeneration (86). This process is essential for optimal thymus regeneration as blocking BMP using a pan BMP inhibitor significantly impairs thymus repair (86). Interestingly, thymic endothelial cell regulation of TEC and thymus recovery may have therapeutic implications. Indeed, intravenous administration of thymic endothelial cells that were expanded *in vitro* (exEC) significantly improved thymus regeneration upon damage with exEC reported to home to the thymus and potentially exert this effect intrathymically (86). Further therapeutic potential was established by a follow up study which found that administering zinc increased the thymus regeneration and TEC proliferation (87). Zinc supplementation simultaneously increased endothelial cell numbers and induced increased expression of BMP4 to significantly promote thymic reconstitution (87). Interestingly, the zinc receptor GPR39 is expressed at steady-state, but expression is increased upon damage suggesting endothelial cells increase their receptiveness to zinc to enhance their regeneration function (87).

### Thymic Endothelium Regulation by TEC

In a similar fashion to bi-directional cellular crosstalk reported to occur between thymic epithelium and mesenchyme, and thymic epithelium and thymocytes, crosstalk may also operate between epithelial and endothelial compartments. Vascular endothelial growth factor (VEGF) is a key regulator of vascular development and has been shown to be key in the thymus endothelial development during neonatal development but is largely dispensable in adults (88). Thymic epithelial cells, while being influenced by thymic endothelial cells during their development, maintenance and regeneration as described above, may themselves have a reciprocal role in regulating endothelial cells. Muller et al. showed that by targeting deletion of VEGF-A to thymic epithelial, thymus vasculature was significantly altered. Deleting VEGF-A on TEC resulted in hypovascularization and disruption of the normal vascular distribution within the thymus, highlighting VEGF-A as an epithelial growth factor which is required for normal thymus vasculature development (89). Inhibition of VEGF in neonatal mice also significantly reduces the frequency of endothelial cells within the thymus (90, 91). Additionally, loss of Foxn-1 expression in Foxn-1^Δ/Δ^ embryonic mice (homozygous for hypomorphic Foxn-1^Δ^ allele; model with less severe phenotype than foxn-1^null^ mice) causing significantly reduced VEGF expression which resulted in significant changes to the vasculature within the thymus including reduced vessel integrity and disorganized patterning/distribution (92). These data are interesting as they emphasize how epithelial cells support the development of the vasculature and other studies demonstrate that normal vascular development is essential for the support of thymic epithelial cells, highlighting a level of cross-talk between the two populations to ensure the optimal development of each compartment.

## Non-Epithelial Stroma and Age-Related Thymic Involution

The thymus undergoes drastic age-associated involution which impacts immune function (93, 94). With age, the thymus becomes smaller, T-cell output is diminished and stromal microenvironments change (93, 95, 96). With regards to the latter, a considerable amount of aged thymic space is composed of adipose tissue, which occurs alongside a reduction in the area and organization of the TEC compartment due to cTEC and mTEC loss (97, 98). These impaired cTEC and mTEC compartments are associated with significantly reduced thymocyte development and output in aged thymus (97). In contrast, how thymic NES populations change with age is less well documented, and so little is known about how changes in these cell types may relate to declining age-related thymus function. Relevant to this, fibroblasts represent an increasing stromal population within the aging thymus (95). While the reasons for this are not fully clear, it has been suggested that it may occur as a result of increased epithelial-mesenchyme transitioning (EMT) occurring during aging, which causes increased fibroblasts, which may then also transition further into adipocytes (99). Consistent with this, thymic mesenchymal cells possess the ability to differentiate into adipocytes from thymosphere-forming cells (37).

Interestingly, it has also been shown that adipocytes accumulate in the PVS within the aged thymus (100). While the potential functional significance of this is not clear, it raises interesting questions, including how might increased adipocyte frequency at this site influence thymus entry and/or emigration? As the PVS is a site of mature thymocyte egress, alterations in these regions may contribute to any reduced thymic output already caused by thymocyte development defects linked to reduced/altered TEC. In addition, it has been shown that thymocyte development is supported by NES, for example mKitL production by endothelium, or IL-7 presentation by mesenchymal ECM components (22, 76). Thus, age-related alterations in NES may impair thymopoiesis by limiting these processes. Interestingly, IL-7 is reduced with age and may contribute to reduced thymopoiesis seen with aging (101). Also, while IL-7 is a TEC product (102) it may be interesting to consider whether changes in thymic mesenchyme in an aged thymus result in less effective presentation of this cytokine, as compared to their counterparts in a younger thymus. In sum, it is important that as our understanding of the characteristics and functions of the NES compartment continues to expand in the steady state thymus, it should also be applied to investigations of disease and aging.

## Concluding Remarks

The non-autonomous nature of intrathymic T-cell development, and the subsequent requirement for thymocyte-extrinsic signals from thymic stromal cells, is well established. Of the diverse stromal cell types that contribute to cortical and medullary microenvironments, thymic epithelial cells (TEC) have been the predominant focus of study over many years. This is likely due at least in part to their fundamental importance in shaping the functional properties of αβ-T-cells produced in the thymus. Indeed, TEC populations control MHC restriction and determine the specificity of the T-cell receptor repertoire by both positive and negative selection events. In addition to the importance of TEC biology, non-epithelial stromal cells (NES) have also been a focus of study, but to a significantly lesser extent. However, NES such as mesenchyme and endothelium have been implicated in key aspects of thymus function. For mesenchyme, their influence on T-cell development can operate by multiple mechanisms. Thus, mesenchyme-derived signals can either target developing thymocytes directly (22, 23), or indirectly by regulating TEC function (14, 16, 32). This fits well with the nature of the thymus epithelial-mesenchymal organ and highlights the need for further study of NES-TEC and NES-thymocyte interactions. Relevant to this, the recent identification of newly defined mesenchyme subsets within NES should facilitate a better understanding of their importance.

For endothelial populations within NES, growing evidence supports their importance in controlling cellular trafficking into and out of the thymus. As the efficacy of these processes can act as rate-limiting steps in T-cell production (51, 65, 67, 74, 76, 79), again a better understanding of the endothelial subsets that contribute to intrathymic vessels for both thymus entry and exit is an important goal of future research. Finally, that endothelial cells also play a role in the regeneration of the thymus following damage (86) opens a new chapter in the relevance of these cells in controlling thymus function in health and disease.

## Author Contributions

The review was written by KJ and was revised by WJ and GA. All authors contributed to the article and approved the submitted version.

## Funding

Work in the laboratory is funded by the Medical Research Council (MR/T029765/1).

## Conflict of Interest

The authors declare that the research was conducted in the absence of any commercial or financial relationships that could be construed as a potential conflict of interest.
